# Dr. Scheffer C.G. Tseng: A Pioneer in Cryopreserved Amniotic Membrane for Regenerative Medicine

**DOI:** 10.7759/cureus.66872

**Published:** 2024-08-14

**Authors:** Anny M Cheng, Shailesh K Gupta

**Affiliations:** 1 Ophthalmology, Broward Health, Fort Lauderdale, USA; 2 Ophthalmology, Florida International University, Herbert Wertheim College of Medicine, Miami, USA; 3 Ophthalmology, Specialty Retina Center, Deerfield beach, USA; 4 Ophthalmology, Broward Health, Fort lauderdale, USA

**Keywords:** biographies, regenerative medicine advanced therapy, prokera, historical vignette, cryopreserved amniotic membrane, amniotic membrane transplantation

## Abstract

Dr. Scheffer Chuei-Goong Tseng is widely recognized as a pioneer in the development and application of cryopreserved amniotic membrane therapy. Dr. Tseng has completely revolutionized the management of ocular and various diseases through the success in the study of regenerative medicine, specifically through the human amniotic membrane. He has turned innovative scientific discoveries into products that contribute to many medical fields, including ophthalmology, orthopedics, oral and maxillofacial surgery, dermatology, and wound care. This review article explores Dr. Tseng’s background, career, and significant contributions to regenerative medicine, with a particular focus on the impact of cryopreserved amniotic membrane technology.

## Introduction and background

Amniotic membrane introduction

The main purpose of this article is to highlight the indisputable contribution of Dr. Scheffer C.G. Tseng to regenerative treatment through the use of the birth tissue - human amniotic membrane (AM). The first reported clinical application of AM was for skin transplantation in 1950 [1]; however, it wasn’t until 1995 that Dr. Tseng and his colleague J.C. Kim initially reintroduced AM transplantation for ocular surface transplantation in a rabbit limbal stem cell deficiency model [2]. A variety of plausible mechanisms had been suggested to elucidate the mechanism of action of AM transplantation by 2004 [3].

Histologically, the AM is composed of a simple epithelium, a dense basement membrane, and an avascular stroma that is abundant in hyaluronic acid (HA) [4]. Its relative thickness ranges from 20 to 150 µm. AM also contains growth factors, such as epidermal growth factor (EGF), basic fibroblast growth factor (FGF), transforming growth factor (TGF), hepatocyte growth factor, keratinocyte growth factor, and nerve growth factor (NGF) [5]. Both the AM and umbilical cord (UC) share the same cellular origin with the fetus. The UC consists of the AM as its outer layer, with umbilical vessels located inside that are embedded inside a loose, proteoglycan-rich substance known as Wharton’s jelly. In addition to the presence of collagen, fibronectin‑I, lumican I, glycosaminoglycans, chondroitin/dermatan sulfate proteoglycans, and mesenchymal stem cells, Wharton’s jelly contains many growth factors including basic FGF, EGF, and TGF [6].

After nearly a decade, his laboratory has been dedicated to the pursuit of the key molecular candidate that is responsible for the therapeutic effects of the AM. His research team isolated a matrix component named heavy chain (HC)-hyaluronic acid (HA)/pentraxin 3 (PTX3) from a water-soluble extract of the AM. HC-HA/PTX3 is a distinctive matrix that is prevalent in the birth tissue, including the AM and UC. This complex consists of high molecular weight HA covalently linked with HC1 from inter-α-trypsin inhibitor and further bound to PTX3 [7,8]. A variety of biological activities in many cell types are coordinated by HC-HA/PTX3.

HC-HA/PTX3 has been demonstrated to induce apoptosis in active neutrophils and macrophages while having no effect on resting cells. It selectively induces the polarization of M2 (anti-inflammatory) macrophages and enhances the active phagocytosis of apoptotic neutrophils to modulate inflammation and effectively prevent any further pro-inflammatory reactions [8,9]. HC-HA/PTX3 exerts a broad-spectrum anti-inflammatory effect not only on neutrophils and macrophages but also on lymphocytes, hence spanning from innate to adaptive immune responses. The adaptive immune in vitro and in-vivo studies showed that HC-HA/PTX3 suppressed Th1 CD4+ cells and promoted the expansion of Tregs to downregulate alloreactive responses to suppress corneal or lacrimal allograft rejection following subconjunctival injection of HC-HA/PTX3 [10,11].

HC-HA/PTX3 also has an anti-scarring effect by suppressing TGF‑β signaling and inhibiting profibrotic and scarring. It has been demonstrated in vivo that HC-HA/PTX3 has anti-scarring properties by reducing excessive inflammation and fibrosis of chronic murine graft versus host disease in the lacrimal gland and conjunctiva, as demonstrated by weak Mallory staining and a lack of infiltration of CD45+CD34 + collagen I + CXCR4 + fibrocytes [11]. The anti-scarring effect of HC-HA/PTX3 to reduce keratocyte (fibroblast) necrosis and reduce differentiation into myofibroblasts (pro-scarring cells) has also been proven in many pre- and clinical studies [12-14]. Urgent AM treatment to decrease the risk of scarring and visual sequelae has been described as a standard treatment guideline for the acute ocular manifestations of Stevens-Johnson syndrome and toxic epidermal necrolysis [15]. A healthy environment for cell adhesion, growth, and differentiation is fostered by the AM's collective anti-inflammatory and anti-scarring effects.

In addition, AM acts as a surrogate niche to support limbal epithelial stem cells [16]. HC‑HA/PTX3 suppresses canonical Wnt but activates noncanonical Wnt (planar cell polarity) and BMP canonical signaling in both limbal niche and epithelial cells to preserve the quiescence of limbal epithelial stem cells [17,18]. It is a special substrate employed to enhance the proliferation of limbal epithelial stem cells while preserving stem cell quiescence, self-renewal, and fate determination [16,19].

More recently, AM has been demonstrated to facilitate corneal nerve regeneration [20-22], and the restoration of normal nerve function is proposed as a potential mechanism for pain relief [21,23]. AM’s composition, which includes HC-HA/PTX3, NGF, and neurotrophins (NTs), which are important for neuronal survival and development [5], can directly contribute to its beneficial effects on corneal nerve regeneration. Alternatively, AM’s anti-inflammatory and anti-scarring properties, which indirectly promote a pro-regenerative environment similar to fetal scarless healing, can explain these benefits. In addition to ophthalmology [24-26], the regeneration qualities of AM have also been shown in spina bifida [27,28], diabetic foot ulcers [29,30], surgical reconstruction of limbs [31], radical prostatectomy [32], maxillofacial wounds [33], and complete skin defect [34]. The potential role of HC-HA/PTX3 in supporting regeneration is being investigated further in various medical fields. It is conceivable that regeneration therapy utilizing AM might potentially serve as a novel biological approach to not only restore the health of the ocular surface but also address unmet medical requirements in many degenerative disorders that extend beyond the ocular surface.

## Review

Early life and education

**Figure 1:**
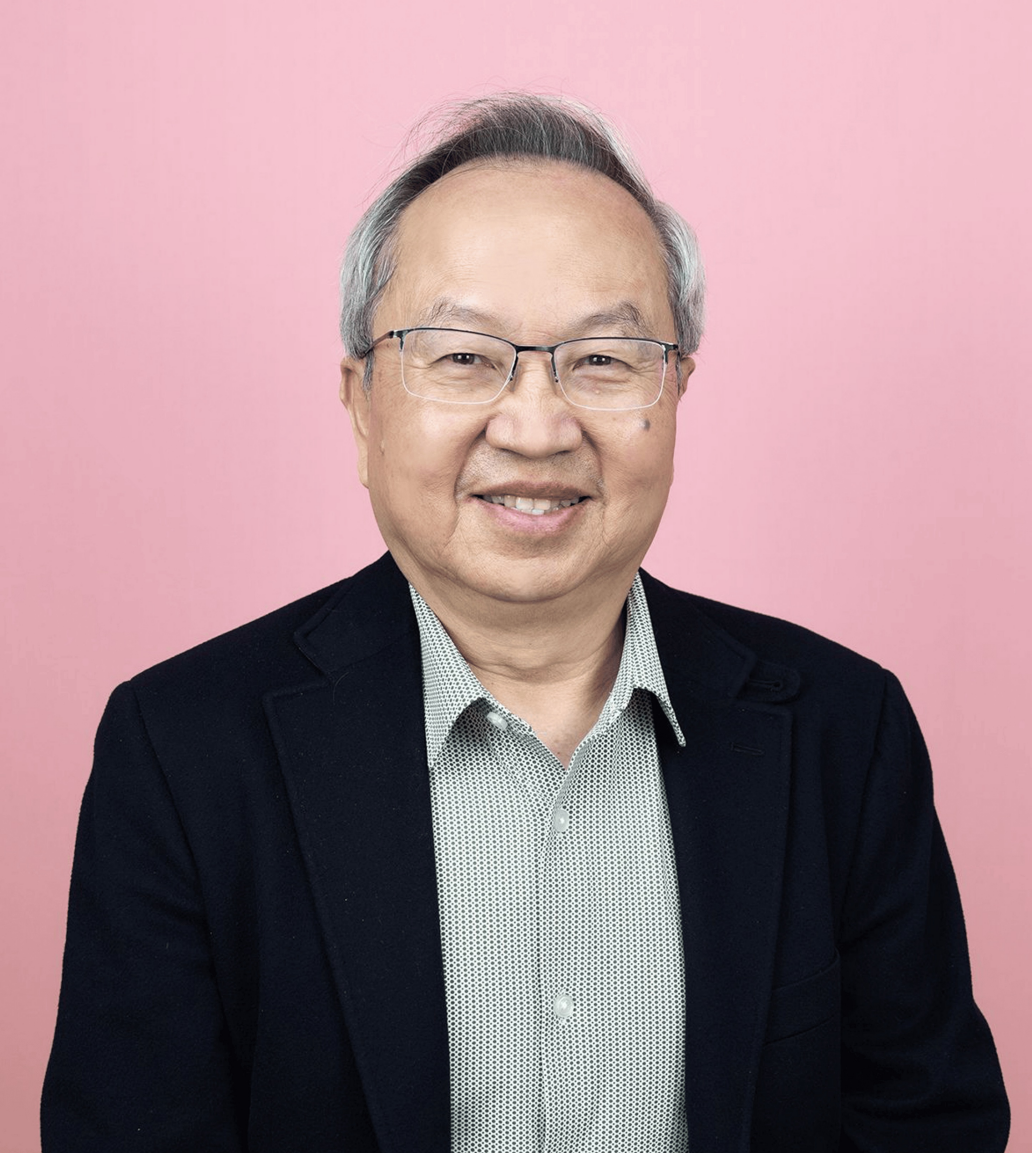
Photo of Scheffer C.G Tseng, MD, PhD A Pioneer in Cryopreserved Amniotic Membrane for Regenerative Medicine Image source: Biotissue, Inc. Original photo used by permission - We thank you Biotissue, Inc.

Dr. Scheffer Tseng was born in Taiwan, where he developed an early interest in science and medicine. He began his academic path at National Taiwan University, the most prestigious national institution in Taiwan, where he obtained his medical degree (1978). Dr. Tseng pursued advanced medical research, which brought him to the United States. He successfully earned a PhD in Experimental Pathology from the University of California San Francisco (1981). He completed his residency in ophthalmology at the Wilmer Eye Institute at Johns Hopkins (1984), and his fellowship in cornea and external diseases at Massachusetts Eye and Ear Infirmary, Harvard Medical School (1986). Prior to co-founding TissueTech/BioTissue Inc, Dr. Tseng was a Charlotte Breyer Rodgers Chair Professor at Bascom Palmer Eye Institute (till 2002), which is consistently ranked as one of the top ophthalmology programs in the world (Figure 1).

Pioneering work with cryopreserved amniotic Membrane (CAM)

Upon completing his residency, Dr. Tseng continued his fellowship in corneal and external disease. This period was formative in shaping his research interests and clinical expertise. Dr. Tseng developed a keen interest in the potential therapeutic applications of biological tissues, which eventually led to his pioneering work with AM.

Recognizing the unique matrix HC-HA/PTX3 in AM and associated beneficial healing and tissue regeneration properties, Dr. Tseng hypothesized that cryopreserved AM (CAM) could be used effectively in various medical treatments. In the early 1990s, Dr. Tseng at Bascom Palmer Eye Institute began his research into the use of CAM in ocular surface reconstruction. In order to preserve the biological and structural characteristics of the fresh AM tissue while preventing the spread of potential infectious diseases in fresh tissue, appropriate tissue preservation and storage technique is crucial. Cryopreservation is the process of storing and transporting tissue at low temperatures. This causes the live cells to lose their vitality, but the tissue keeps its inherent structural and biological features [4]. These cryopreserved AM and UC can be stored for a duration of 2 years within a temperature range of -80°C to 4°C. In contrast, another technique for processing is dehydration, which involves applying heat to the tissue to eliminate moisture. A comparative laboratory study showed that the active HC-HA/PTX3 complex is exclusively retained by cryopreservation [4], and previous studies have demonstrated comparable results when using fresh and cryopreserved AM [35].

Establishment of TissueTech and the BioTissue, Inc.

In 1997, Dr. Scheffer C.G. Tseng co-founded BioTissue Inc (Miami, FL, USA), a biotechnology company pioneer of the development of regenerative medicine products based on human AM and UC. In 2001, TissueTech Inc was founded as the parent entity of BioTissue. Cryopreserved AM (AmnioGraft; BioTissue, Miami, FL, USA) was introduced in 1997 and classified as a 361‑human cell/tissue product in 2001 by the Food and Drug Administration (FDA) for the purpose of ocular surface reconstruction. The AM, thickness of 75 to 150 µm, is attached to a nitrocellulose carrier sheet with the sticky stroma side, which is then detached during transplantation. His CAM transplantation was revolutionary, offering new hope for patients with severe ocular conditions that were previously difficult to treat. CAM transplantation could effectively treat various complex ocular surface disorders, such as band keratopathy, chemical burns, ulcers, basement membrane dystrophy, limbal stem cell deficiency, Stevens-Johnson syndrome, graft versus host disease, persistent epithelial defects, neurotrophic keratitis, dry eye disease, Sjogren syndrome, filamentary keratitis, HSV keratitis, and retinal break or hole. Later in 2004, an office placement of self-retained cryopreserved AM (PROKERA; BioTissue, Miami, FL, USA) was approved by the FDA as a Class II medical device under 510(k) #K032104 to prevent suture-related inflammation. PROKERA consists of a piece of CAM mounted on a polycarbonate ring. It can be easily placed on the eye, where it delivers its therapeutic benefits directly to the affected area. This innovative product has significantly advanced the field of ophthalmology and has become a standard treatment for many eye diseases [15,24,36-41]. In 2011, a cryopreserved UC (AmnioGuard, BioTissue, Miami, FL, USA), was introduced and widely utilized to restore the ocular surface following the removal of melanoma, carcinoma, and pterygium, to address socket contracture, orbital implant exposure, forniceal contracture, and entropion repair, and as a barrier graft for glaucoma shunt tubes [42].

Recently, Dr. Tseng and his research team have developed a PROKERA-like but ringless CAM (CAM360 AmnioGraft™, BioTissue, Miami, FL, USA) that is the first and only hydrated CAM developed to optimize comfort and exhibit HC-HA/PTX3 properties for ocular use. In ophthalmology, many amniotic-derived products are currently available. Efficient methods for preserving and storing tissues are crucial for retaining the biological characteristics of the fresh AM. Cryopreserved AM products have been demonstrated to maintain the unique matrix HC-HA/PTX3 complex to preserve the natural therapeutic biological properties of anti-inflammatory, anti-scarring, and regenerative function [4].

Broad medical applications

While Dr. Tseng’s work initially focused on ophthalmology, the potential applications of CAM extend far beyond eye care. His research and development efforts have demonstrated the efficacy of CAM in many other medical fields. Studies have shown that CAM can significantly improve healing rates in chronic and non-healing wounds, such as diabetic foot ulcers [29,30,43], vascular [44] , and pressure ulcers [45]. In addition, CAM has been reported to prevent contractures following profound burn injuries, hence promoting functional recovery [46]. Tendon injuries often require prolonged healing periods and can lead to significant functional impairment. CAM provides a natural scaffold that supports tissue regeneration and reduces the risk of infection. CAM wrapped around the tendon repair site resulted in quicker function and qualitatively better tendon healing with a decrease of inflammation [47]. Intra-articular injection of AM-UC (CLARIX FLO, BioTissue, FL, USA) has been successfully utilized to promote healing in various orthopedic indications [48,49] and reduce pain caused by facet joint syndrome [50]. Its anti-inflammatory and regenerative properties could help to improve healing outcomes in musculoskeletal injuries. CAM has been used to promote mucosal healing and accelerate epithelialization in oral mucosal defects resulting from surgical excisions or trauma [51]. In addition, CAM promotes the regeneration of gum tissue and provides a protective barrier during the healing process in periodontal surgery to treat gingival recession [52]. Neural injuries can lead to severe and often irreversible damage, resulting in paralysis and loss of function. CAM has been used as a homograft for dural reconstruction in myelomeningocele repair to prevent infection, minimize neural tissue damage, and reduce mortality [53].

From chronic wounds and burn injuries to orthopedic, oral, and maxillofacial surgery, and neurological applications, CAM has demonstrated its ability to enhance healing, reduce complications, and improve patient outcomes. As research continues to uncover new applications and refine existing techniques, the use of CAM in medicine is likely to expand further. Its effectiveness makes it a promising option for clinicians seeking advanced treatments for challenging medical conditions.

Recognition

Dr. Tseng’s significant contributions to medicine have been widely recognized. He has received numerous awards and honors throughout his career. Dr. Tseng has integrated his extensive knowledge in clinical services, teaching, and research. He has authored over 380 peer-reviewed publications and received numerous invitations to present at international conferences and symposiums. In addition to his research and clinical work, Dr. Tseng is committed to giving back to the medical community. He has mentored countless medical students, residents, and over 80 fellows, many of whom have gone on to make their own significant contributions to medicine. Dr. Tseng is also involved in various philanthropic activities, supporting initiatives that aim to improve access to medical care and advance medical research.

Dr. Tseng continues to be actively involved in research and innovation. His ongoing work aims to explore new applications for CAM and other regenerative medicine technologies. As the understanding of the biological mechanisms underlying CAM’s therapeutic effects continues to evolve, it is likely that new medical applications will emerge. Dr. Tseng’s legacy is defined by his unwavering commitment to improving patient care through innovation and research. His pioneering work with CAM has transformed the landscape of medical fields, provided new treatment options and improved outcomes for countless patients. As a visionary leader in regenerative medicine, Dr. Tseng’s contributions will continue to impact the medical community for years to come.

## Conclusions

Dr. Scheffer Tseng’s pioneering work with CAM has had a profound impact on the field of regenerative medicine. From his early research in ocular surface reconstruction to the development of AmnioGraft, PROKERA, CAM360, and the broad application of CAM in various medical fields through different AM products, Dr. Tseng’s contributions have revolutionized patient care and opened new avenues for treatment. His dedication to research, innovation, and mentorship has solidified his legacy as a true medical pioneer. As the medical community continues to explore the potential of regenerative medicine, Dr. Tseng’s work will undoubtedly serve as a foundation for future advancements.
